# Geriatric Residents' Perceptions of Training for Good Dying: A Qualitative Case Study in Colombia

**DOI:** 10.1111/tct.70414

**Published:** 2026-04-16

**Authors:** Cristian Camilo Llano Ceballos, Nelson Julian Quiroga Laverde, Martha Patricia Montoya Montoya, Karin Natalia Perdomo Nuñez

**Affiliations:** ^1^ Unidad de Geriatría Hospital Universitario San Ignacio Bogotá Colombia; ^2^ Facultad de Medicina, Departamento de Medicina Interna Pontificia Universidad Javeriana Bogotá Colombia; ^3^ Universidad Antonio Nariño Bogotá Colombia; ^4^ Hospital Universitario Mayor Méderi Bogotá Colombia; ^5^ Clinica Infantil Colsubsidio Universidad del Rosario Bogotá Colombia

**Keywords:** death, geriatrics, good dying, medical education, residents, support

## Abstract

**Introduction:**

Death is a universal phenomenon influenced by cultural, social and religious factors. Despite its relevance to medical practice, geriatric residents often lack formal education on how to support patients in the dying process.

**Methods:**

A qualitative case study design was conducted at a tertiary care hospital in Bogotá, Colombia. Twelve first and final‐year medical residents were interviewed. The interviews were recorded, transcribed and analysed through triangulation with theoretical references and curricular documents from the geriatric residency programme.

**Results:**

Training in ‘good dying’ was found to be variable, encompassing four core components (symptom control, communication, bioethics and psychosocial support). Key learning strategies identified included theoretical review, artistic representation, role‐playing and reflection spaces. Teaching was strongly influenced by modulating factors such as the role models provided by faculty and senior residents and the deficiency of an explicit curriculum. Near‐peer learning and faculty role modelling emerged as central influences shaping residents' approaches to death and good dying, often outweighing formal curricular content.

**Conclusions:**

Training in good dying within geriatric residency was characterised by reliance on experiential and relational learning, with senior residents acting as key mediators of the hidden curriculum. Making these educational mechanisms explicit may support more coherent and intentional approaches to end‐of‐life training. The explicit inclusion of promoting good dying in the academic curriculum is recommended. This would improve student training, optimise learning outcomes and ensure more comprehensive and humane care for patients at the end of life.

## 1 Introduction

In healthcare institutions, residents strengthen care teams but face death with limited training and support [1]. This lack of training leads to negative emotions such as fear and anxiety, which affect professional development and the quality of care provided [2]. End‐of‐life education is essential for improving communication and humanising care.

Despite growing recognition of the importance of education on end‐of‐life issues, academic literature and educational strategies remain inconsistent and are often confined to the hidden curriculum [3]. However, this landscape is gradually shifting, with increasing openness towards teaching about death. Although the definition of good dying is not standardised, it can be summarised as providing a positive, dignified experience for the patient.

Rather than conducting a systematic comparison, the concept of ‘good dying’ was compiled through narrative review considering cultural similarities from an Ibero‐American perspective where family‐centred decision‐making and religious values predominate over individual autonomy. We reviewed key countries with similar socio‐cultural backgrounds that have implemented educational initiatives on the pedagogy of death. Countries such as Spain, Mexico, Argentina and Colombia have initiated diverse pedagogical efforts, ranging from grief standardisation and cinema‐based empathy training to artistic practices and the teaching of therapeutic proportionality [4, 5, 6, 7]. Nevertheless, despite these isolated efforts, end‐of‐life education in Latin America remains largely marginal [8, 9]. Other Western countries have developed pedagogical strategies to address death in medical education, including workshops and simulations [4]. In the United States, end‐of‐life education has evolved into a comprehensive approach focused on communication with patients and families [10].

This cultural nuance creates unique tensions between clinical protocols and patient–family expectations, highlighting the need to provide culturally sensitive training to geriatric residents, who care for the frail and terminal populations [11].

Despite these advances, education for ‘good dying’ lacks global standardisation, as it is heavily influenced by regional customs. In the local Colombian scenario, regulations recognise dignified death but do not address educational needs. Research indicates an absence of end‐of‐life training in medical curricula, relegating it to implicit learning and generating overly procedural approaches to medical education [7]. Consequences of this deficiency include avoidance of terminally ill patients, thanatophobia (fear of death), compassion fatigue and distrust of health professionals [11].

This study investigates the training of postgraduate geriatric residents in Colombia—specifically those enrolled in one of the country's five geriatrics programmes—in crucial aspects of facilitating good dying and providing patient accompaniment. Furthermore, it explores residents' perceptions of the training experience and its impact on patient care. It is critical to understand that unlike other specialties (where death may be viewed as a clinical failure), in geriatric medicine, the accompaniment of decline is a core competency. Given the specialty's proximity to the end of life, ensuring the quality of these skills is crucial. From the authors' perspective, the focus on a specific geriatric residency programme provides a deep, contextualised understanding of training perceptions and experiences and helps bridge the international and multicultural knowledge gap.

*Unlike other specialties (where death may be viewed as a clinical failure), in geriatric medicine, the accompaniment of decline is a core competency.*

Therefore, this qualitative case study aims to answer the following research question: What are geriatric residents' perceptions of the formal and informal training components used to teach accompaniment in ‘good dying’, and what is the resulting impact of this training experience on patient care in this context?

## 2 Materials and Methods

A qualitative case study design was employed to examine the geriatric residency programme at a tertiary hospital in Bogotá, Colombia. This approach allowed for an exploration of ‘good dying’ training as a bounded system, integrating curricular analysis, resident perceptions and clinical practice [12]. This design is particularly effective for identifying the disjunction between formal and ‘hidden’ curricula in high‐pressure environments.

The study population comprised all 14 residents of the Geriatrics Postgraduate Program at Pontificia Universidad Javeriana, rotating in the Emergency Department and Inpatient Unit of San Ignacio University Hospital under specialist supervision, encompassing the entire available population. Twelve residents participated; two declined. Inclusion of both early‐ and advanced‐year residents ensured data richness, and thematic saturation was reached when no new themes emerged.

Data collection involved semi‐structured interviews, which were recorded, transcribed and validated by participants. A checklist adapted from Gay, Mills and Airasian [13] ensured methodological consistency. Simultaneously, curriculum documents were reviewed for formal instruction components.

Thematic analysis was performed using Atlas.ti. Coding followed an iterative deductive‐inductive process, triangulated across four researchers and compared with theoretical propositions to ensure credibility. Regarding reflexivity, the research team comprised four members with diverse clinical and academic backgrounds. The principal investigator, a geriatrician affiliated with Pontificia Universidad Javeriana and Hospital Universitario San Ignacio, was the only team member with direct involvement in the institutional context under study. The remaining three researchers—a professional nurse, a family medicine specialist and a paediatric palliativist—were affiliated with different institutions and had no prior personal or academic contact with the residents interviewed. To minimise the potential influence of insider positionality on data collection, interviews were conducted exclusively by the nurse and the family medicine specialist, ensuring that participants had no prior relationship with their interviewers. Throughout the study, all four researchers engaged in iterative group reflection during team meetings, actively bracketing individual perspectives and clinical assumptions to prevent these from shaping data interpretation. Consensus on coding and thematic categories was reached through structured discussion and triangulation across all four researchers, reinforcing the credibility and trustworthiness of the findings.

The study complied with the Declaration of Helsinki and received approval from the institutional research board. Participation was voluntary, and confidentiality was maintained through coded identifiers.

## 3 Results

### 3.1 Category Analysis

The transcripts of the initial interviews were coded inductively by four researchers using the aforementioned software. They then met to compare and reach consensus through an iterative deductive–inductive process, reconciling emergent codes with theoretical propositions. Once obtained, the information was organised into three categories: components of teaching good dying, strategies for teaching good dying and factors modulating teaching good dying. Team meetings were held to organise categories and perform coding. Thematic analysis ended when no new themes were identified, indicating saturation and producing the following results. For better understanding, categories are defined in Table 1.

**TABLE 1:** Categories and subcategories of good dying teaching components, strategies and modulating factors.

Category	Subcategories
Components of teaching (CT)	Symptom control (CT‐SC); communication (CT‐C); bioethics (CT‐B); psychosocial aspects (CT‐PS)
Strategies for teaching (ST)	Theoretical review (ST‐TR); artistic representation (ST‐AR); role‐playing (ST‐RP); reflection spaces (ST‐RS)
Modulating factors (MF)	Faculty role (MF‐TR); residents as teachers (MF‐RRT); personal practices (MF‐PP); explicit curriculum (MF‐EC); previous experiences (MF‐PE)

Once the transcription, categorisation and coding exercises were completed, a review of supporting documentation was conducted, including the ‘Master Document for Requesting the Renewal of the Qualified Registry – Postgraduate Program in Geriatrics, Pontificia Universidad Javeriana 2018’ [14]. The analysis was linked to reports in the literature, yielding the main findings, which are presented below, organised by categories and their respective subcategories. Interviewee statements appear in quotation marks.

#### 3.1.1 Components of Teaching Good Dying (CT): The ‘What’ of Training

This category addresses the perceived content or components from formal and informal instruction. Residents perceived that training, when it occurred, successfully covered a broad scope encompassing physical, emotional, social, family and spiritual aspects of care.

##### 3.1.1.1 Symptom Control

Residents perceived symptom control as a core component of their training, with emphasis on palliative sedation and ongoing assessment of physical distress. However, the data revealed a gap in the perceived impact on patient care: Despite receiving instruction, residents reported uncertainty about comprehensive palliative care delivery—a finding consistent with the broader literature [15, 16], which indicates that formal training alone does not translate into full clinical confidence. This suggests that current training, while covering the ‘what,’ is not translating into optimal performance. One interviewee mentioned:

In geriatrics, we learned a lot about assessing patients at the end of life, with ongoing support. (E12R49)

Furthermore, although the curriculum includes symptom management in palliative care, no other learning scenarios were evident in the reviewed document [14].

Learning this skill is important because of the previously described poor symptom control at the end of life, as reported by patients and families in the literature. First‐year residents may have lower confidence in providing this relief compared to final‐year residents, and this is clearly affected by experience and exposure [15].

##### 3.1.1.2 Communication

Communication is fundamental to the process of good dying, with emphasis on the effective delivery of bad news. According to interviewees, tools such as SPIKES are used to structure the dialogue:

Communication is more relevant to bad news and confirming that the message was understood. (E1R41)

The curriculum mentions subjects such as ‘Communication and Geriatrics’ [14] and the use of the clinical simulation centre, but interviewees did not report using them. Although physicians tend to overestimate their communication skills, some acknowledge the following deficiencies:

There are difficulties in clarifying medical terms and transforming the message so that it is understood. (E4R42)

Death is commonplace in hospital settings, yet clinicians often struggle to communicate this type of news. Given the scope of practice in geriatric medicine, residents recognising these difficulties during training should raise alarms regarding the need to strengthen these crucial skills. This reinforces the notion that residents often perceive communication as a checklist‐driven procedure (e.g., SPIKES) rather than a relational competency. This can lead to ‘mechanized empathy’ that fails to address the existential depth of the dying process [17].

##### 3.1.1.3 Bioethics

Bioethics instruction addresses ethical dilemmas and end‐of‐life decision‐making. Instruction is provided on clinical ethics and criteria for futile interventions, which some respondents identified as part of their training:

We are given guidelines on considerations for good dying and how to deal with ethical dilemmas. (E12R49)

The curriculum included a clinical bioethics seminar, although this was not mentioned by participants. This illustrates the difficulty of translating complex theory into real‐world clinical scenarios, where principles learned in the classroom fail to manifest as practical tools during the urgency of bedside decision‐making. This gap represents a form of translational ethics, where complex concepts fail to serve as tools at the bedside [18].

##### 3.1.1.4 Psychosocial

The psychosocial component of good dying involves grief and perceptions of death, which are influenced by cultural and social factors. The importance of understanding the patient's environment for decision‐making and treatment adherence is recognised:

It is important to understand the patient's position, the family's position, and the patient's wishes in order to maintain autonomy. (E6R44)

However, the psychosocial component is limited to a single programme out of five existing postgraduate geriatrics programmes and is associated with Western and Colombian perceptions of death and good dying. The results imply that psychosocial training heavily relies on the resident's cultural intuition rather than a structured andragogical model, making it context dependent. This makes the quality of humanistic care susceptible to the individual resident's background [8].

#### 3.1.2 Strategies for Teaching Support in Good Dying (ST):The ‘How’ of Training

Education about death has become increasingly important in medical education [6]. This process should include both formal and informal elements and address cognitive, affective, behavioural and evaluative dimensions. Teaching about good dying should be linked to learning experiences that promote positive attitudes about death, the dying process, grief and caring for those affected by loss [16].

##### 3.1.2.1 Theoretical Review

Teaching about good dying through theoretical review includes lectures, seminars and topic reviews. The goal is strengthening professional competencies, which include knowledge, practice and self‐competence [16]. However, education takes place in mono‐professional and self‐regulated settings, which lead to differences in student exposure. Examples from respondents include:

After accompanying end‐of‐life and good dying, I provide reinforcement with topic reviews and guides. (E6R44)

During my palliative care rotation, we cover symptom management and end‐of‐life in seminars. (E12R49)

Although the importance of learning is correlated with the clinical setting, there is no standardised programme for teaching this content [16, 19]. The master document for the geriatrics specialty also does not define clear teaching strategies, although it includes the palliative care rotation as a requirement [14]. This provides evidence regarding the lack of standardisation of training even within the same programme, where experience can vary between residents.

##### 3.1.2.2 Artistic Representation

Arts such as film, music and drama are used to encourage reflection on good dying [20, 21]. The geriatrics programme encourages the integration of arts into training [14]. As residents noted:

We created open journals, fanzines, and a Mexican‐inspired altar of the dead. (E4R42)

I have participated in weaving activities and reflections on movies and books. (E4R42)

However, not all respondents were aware of these learning strategies:

I know there are film forums on these topics, but I have not participated. (E6R44)

Intrinsic motivation regulates learning associated with artistic representations but reported not having participated, suggesting that motivation to engage with these strategies is not uniformly distributed among residents [20, 22].

##### 3.1.2.3 Role‐Playing

Role‐playing is a pedagogical strategy in which students simulate clinical situations guided by a tutor, allowing them to improve their communication and decision‐making skills [23].

Although not described in the geriatrics programme document, some interviewees noted:

Acting: one is the doctor and the other the patient with terminal cancer. (E6R44)

A doctor gave us a workshop on how to deliver bad news through role‐playing. (E7R45)

As reported in the literature, this methodology improves communication and clinical performance [23], and simulation provides a controlled environment that fosters the development of skills without exposing patients to adverse outcomes.

##### 3.1.2.4 Reflection Spaces

Reflection spaces are fundamental in medical training, allowing analysis of clinical and emotional experiences at the end of life. Within the geriatric residency programme, meaningful learning scenarios are encouraged [14]. Some notable spaces include:

- Balint group: Meetings with psychiatrists to discuss emotionally impactful cases.
  The Balint group at Javeriana University allows for reflection on end‐of‐life cases. (E4R42)
- Café Converso: Facilitated by a psychiatrist, where residents share experiences and tools for dealing with end‐of‐life support.
  In Café Converso, we discussed end‐of‐life care. (E4R42)

Reflection strengthens the integration between technical training and emotional management, promoting autonomy and a sense of belonging to humanity [24].

#### 3.1.3 Modulating Factors (MF): Context Influencing Perception and Impact

Teaching support for good dying is influenced by factors such as the environment, cultural practices, social values and patients' personal conditions [24]. For effective teaching, it is important to integrate affective, behavioural and evaluative aspects with technical ones [16].

##### 3.1.3.1 Teaching Role

The teacher plays a pivotal role in medical education, not only by transmitting knowledge but also by demonstrating how to act, evaluate and communicate in death‐related scenarios [25]. However, the lack of a formal approach to death education and the perception of death as taboo limit this teaching [26].

- Teaching gap: Residents learn primarily by observing others in practical scenarios, with initial guidance but without ongoing support [1].
- Positive and negative examples: Some faculty members explain death with empathy and accessible metaphors, facilitating family understanding. Others, however, demonstrate communication deficits that, in contrast, also generate learning [27].

Because death is a unique experience for everyone, even if the teacher has experience with these scenarios, their personal perspective and beliefs influence their students' learning. Consequently, the faculty's role is perceived as a double‐edged sword. The lack of a standardised approach forces residents to filter and adopt empathetic or avoidant behaviours based on their role models [28].

##### 3.1.3.2 Residents' Role as Teachers

The senior resident acts as a teaching facilitator, transmitting knowledge and values in clinical practice [29]. However, the lack of formal training means that this occurs spontaneously through tradition and experience [30].

Training by residents who lack formal pedagogical preparation can be a limiting factor in ensuring effective learning. The role of senior residents (R3/R4) as primary gatekeepers of the hidden curriculum is a critical finding. For junior residents (R1), the senior's behaviour at the bedside carries more weight than formal bioethics lectures. When a senior resident evades conversation about death, they implicitly teach that avoidance is a valid professional coping mechanism, thereby perpetuating a cycle of ‘clinical silence’ and taboo across generations of trainees.

*When a senior resident evades conversation about death, they implicitly teach that avoidance is a valid professional coping mechanism, thereby perpetuating a cycle of ‘clinical silence’ and taboo across generations of trainees.*

##### 3.1.3.3 Personal Practices

Residents use personal strategies to manage emotional burden and improve patient care, including:

- Incorporating elements that are meaningful to the patient (music, pets and movies).
- Religious or spiritual practices for peace and family support.
- Participation in programmes such as Mindfulness to manage stress [31].

##### 3.1.3.4 Explicit Curriculum

The medical curriculum does not address teaching about death in a structured way, focusing more on technical skills than on end‐of‐life reflection [31]. Although the importance of good dying is recognised in the geriatrics specialty, it is not explicitly developed in the curriculum [13].

##### 3.1.3.5 Previous Experiences

Previous experiences (personal and professional) are a strong modulating factor that shapes residents' perceptions. Residents' prior encounters with death allow them to distinguish between a humane end of life and one with futile and invasive measures [32, 33]. This experience directly impacts their ability to make critical, humanistic decisions in patient care. The key educational insights derived from the results presented are summarised in Table 2.

**TABLE 2:** Educational insights derived from residents' perceptions of training in good dying.

Key educational insight	Interpretation for clinical teaching	Considerations for programme development
Predominance of informal learning	Residents rely primarily on observation and experiential learning rather than formal instruction to navigate end‐of‐life situations	Explicit acknowledgment of informal learning processes may help educators align intended and enacted curricula
Central role of senior residents	Senior residents function as near‐peer role models, shaping attitudes towards death, communication practices and emotional engagement	Faculty may consider supporting senior residents in their teaching role through guided reflection or mentorship
Fragmented exposure to teaching strategies	Reflective spaces, role‐playing and artistic approaches are valued but inconsistently accessed	Greater curricular integration could reduce variability in learning opportunities across residents
Proceduralised communication	Communication tools are sometimes perceived as checklist‐driven rather than relational	Educators may explore teaching approaches that emphasise meaning‐making and emotional presence alongside structure
Absence of an explicit curriculum on good dying	Learning occurs through trial, error and personal coping strategies	Defining good dying as a core competency could support more intentional and longitudinal training

## 4 Discussion

This study provides unique qualitative insight into ‘good dying’ training within a geriatric residency, confirming that such education is neither homogeneous nor standardised. This aligns with international trends where, despite high clinical exposure to death, formal training remains inadequate [15, 16]. Our data suggest a significant gap between national policies and actual structured curricular practice at the bedside.

Residents identified four critical pillars—symptom control, communication, bioethics and psychosocial support—yet reported low perception of competence. This misalignment stems from a focus on ‘clinical competence’ at the expense of ‘emotional management’. Although residents utilised self‐management tools like mindfulness and art, the lack of formal, mandatory reflection spaces like Balint groups remains a risk. Without these, we prioritise producing specialists who are technically proficient but emotionally isolated.

A central finding is the overwhelming weight of the informal curriculum. Consistent with socio‐cognitive theory, learning is primarily reflective, experiential and collaborative, yet it lacks structure. Three elements are decisive: role modelling, where faculty influence resident approaches through clinical behaviour rather than lectures; near‐peer learning, where senior residents act as primary facilitators of values; and the risk of distancing, where the informal curriculum may favour emotional withdrawal as a survival mechanism [3]. Without formalising an ‘andragogy of good dying’, programmes risk perpetuating a cycle of neglect where avoidance is misconstrued as ‘professionalism’ [1, 3].

*Without formalising an ‘andragogy of good dying’, programmes risk perpetuating a cycle of neglect where avoidance is misconstrued as ‘professionalism’.*

Furthermore, our findings offer significant implications for medical education across diverse cultural contexts. Although this study was developed in a Latin American setting where family is essential for decision‐making and religious values often predominate, the underlying educational insights particularly regarding the power of the hidden curriculum and near‐peer mentorship are broadly applicable. In individualistic societies, training might focus on legal advance directives; however, in collectivist cultures, the ‘accompaniment’ identified here requires specific training in family mediation. Recognising these nuances allows global institutions to adapt ‘good dying’ competencies into culturally responsive practices.

To move beyond fragmented care, ‘good dying’ must be taught longitudinally rather than in isolated blocks [30, 31, 32, 33, 34]. Strengthening emotional education is not merely a ‘soft skill’ but a clinical necessity for developing humane physicians capable of navigating death without defaulting to emotional withdrawal.

*Strengthening emotional education is not merely a ‘soft skill’ but a clinical necessity for developing humane physicians capable of navigating death without defaulting to emotional withdrawal.*

## 5 Limitations

This study is limited to a single‐site, geriatric‐specific context in Colombia. The findings represent the perceptions of one programme and may not be generalisable. Future research should use mixed methods approaches across various specialties and undergraduate levels to validate these findings and include faculty perceptions.

## 6 Conclusions

This qualitative study highlights the lack of formal standardisation in ‘good dying’ training for geriatric residents. The current reliance on informal and experiential learning results in inconsistent exposure to key competencies. We strongly recommend the formal incorporation of good dying instruction into the postgraduate curriculum, with clear learning objectives that move beyond the hidden curriculum.

Residency programmes should implement structured pedagogical strategies such as artistic representation, dedicated reflection spaces and role‐playing. Furthermore, creating specific training scenarios, such as a ‘good dying response team’, could optimise real‐life learning and faculty–resident interaction. Elevating ‘accompaniment’ to a core clinical competency is essential for ensuring more comprehensive and humane care at the end of life.

## Author Contributions

**Cristian Camilo Llano Ceballos:** conceptualization, methodology, software, writing – review and editing, writing – original draft, formal analysis, data curation, investigation, project administration, validation, resources. **Nelson Julian Quiroga Laverde:** conceptualization, investigation, data curation, formal analysis, writing – review and editing. **Martha Patricia Montoya Montoya:** conceptualization, investigation, data curation, formal analysis, writing – review and editing. **Karin Natalia Perdomo Nuñez:** conceptualization, methodology, supervision.

## Funding

The authors have nothing to report.

## Ethics Statement

The study was reviewed and approved by the Institutional Research Board Committee on April 25, 2024, under the institutional code FM‐CIE 0489‐24. A digital copy of the report, in Spanish, is available in the Supporting Information.

Also, all participants signed an informed consent form prior to their participation in the study. A copy of the informed consent form (in Spanish) is available upon request to the corresponding author.

## Consent

The consents signed by the participants can be sent if the journal requires them as a copy form physical format as a PDF file in native language (Spanish).

## Conflicts of Interest

The authors declare no conflicts of interest.

## Data Availability

The datasets used and/or analysed during the current study (transcript interviews) are available from the corresponding author on reasonable request in native language (Spanish).
